# Key cardiopulmonary exercise testing indicators for predicting the risk of postoperative cardiopulmonary complications in patients undergoing thoracoscopic lung resection

**DOI:** 10.3389/fsurg.2025.1765398

**Published:** 2026-01-13

**Authors:** Nan Yang, Zhihua Shi, Junfeng Liu, Yadong Yuan

**Affiliations:** 1 Department of Functional Diagnosis, The Fourth Hospital of Hebei Medical University, Shijiazhuang, China; 2 Department of Thoracic Surgery, The Fourth Hospital of Hebei Medical University, Shijiazhuang, China; 3 Department of Respiratory and Critical Care Medicine, The Second Hospital of Hebei Medical University, Shijiazhuang, China

**Keywords:** cardiopulmonary complications, cardiopulmonary exercise testing, maximal workload, metabolic equivalent, peak heart rate, peak oxygen uptake, thoracoscopic lung resection

## Abstract

**Objective:**

To explore the predictive value of key preoperative cardiopulmonary exercise testing (CPET) indicators for cardiopulmonary complications following thoracoscopic lung resection.

**Methods:**

Patients who underwent lung resection at the Department of Thoracic Surgery, Fourth Hospital of Hebei Medical University were selected. Information was collected for patients who completed CPET using the incremental exercise protocol. Hospitalization information, postoperative complications and follow-up data were analyzed. Correlations between postoperative cardiopulmonary complications and preoperative CPET indices were analyzed to identify threshold values.

**Results:**

Among 376 thoracoscopic lung resection patients, 52 experienced at least one complication (13.8%). Comparison between the cardiopulmonary complications group (CCP) and no complications group (*N*CCP) revealed significant differences in age, extent of lung resection, and lymph node metastasis (*P* < 0.05). Core CPET indicators including peak heart rate (peak HR), peak oxygen uptake (peak VO2), peak VO2%pred, peak metabolic equivalent (peak MET), and maximal workload %pred were significantly lower in the CCP group (*P* < 0.05). The sensitivity and specificity of peak VO2%pred <70%, peak MET <5, and maximal workload %pred <80% all exceeded 60%, with negative predictive values surpassing 90%. Positive predictive values of peak VO2 < 15 mL/(min·kg), peak VO2%pred <60%, peak MET <4, and maximal workload %pred < 60% exceeded 30%. Using these cutoff values resulted in high diagnostic accuracy with odds ratios of 6.2, 4.0, 4.6, and 3.2, respectively.

**Conclusion:**

Key preoperative CPET indicators effectively evaluate postoperative complication risk in thoracoscopic lung resection patients. Peak VO2, peak VO2%pred, peak MET, and maximal workload %pred are associated with postoperative cardiopulmonary complications.

## 1 Introduction

Lung cancer is a malignant tumor with high incidence and mortality rates worldwide (1, 2). Surgical resection continues to be the recommended treatment for early-stage lung cancer. Thoracic complications are the main type of complication associated with lung resection, with an incidence rate ranging from approximately 10%–49% (3–9). This wide range is primarily attributed to differences in the location and stage of the primary disease, surgical methods, criteria for complication assessment, and analytical methods.

The occurrence of thoracic complications following pulmonary resection can prolong the hospitalization time, affect the long-term prognosis of the patient, and even elevate the risk of mortality. Despite advances in overall medical standards and the widespread application of minimally invasive thoracoscopic surgery, which have reduced surgical trauma, post-thoracic surgical complications remain a significant concern that affect both the recovery of the patient and the effectiveness of surgical treatment. Risk assessment is a standard component of preoperative evaluation for lung resection (10). A robust cardiopulmonary functional reserve is one of the necessary conditions for patients to tolerate lung resection surgery. Therefore, preoperative cardiopulmonary function testing is an important method for evaluating surgical indicators and assessing the risk of postoperative cardiopulmonary complications.

Cardiopulmonary exercise testing (CPET) is an objective, quantitative, and non-invasive method that simultaneously reflects cardiopulmonary metabolism and overall function (11). CPET is regarded as the gold standard for predicting risks associated with thoracic surgery and other major operations requiring complex, high-risk anesthesia management (12, 13). Previous studies investigating the predictive value of CPET indicators for postoperative complications following lung resection have predominantly focused on patients undergoing traditional thoracotomy, which is characterized by a large resection range, severe postoperative complications, and relatively high mortality. Considering the growing prevalence of thoracoscopic surgery, in this study, we focused on patients undergoing thoracoscopic lung resection and their postoperative complications to explore the predictive value of preoperative CPET quantitative indicators for complications after thoracoscopic lung resection.

## 2 Methods

### 2.1 Study subjects

The cases in this study were sourced from the Department of Thoracic Surgery, Fourth Hospital of Hebei Medical University from April 2023 to October 2024. The included patients were diagnosed with pulmonary nodules or lung space-occupying lesions via chest CT and clinically diagnosed with either lung cancer or “pending investigation of the nature of lung space occupying lesions.” Following thoracic surgery evaluation, they were deemed eligible for lung resection surgery. The inclusion criteria were: (1) aged over 18; (2) had undergone bronchoscopy biopsy or percutaneous lung biopsy and been diagnosed with lung cancer by histopathology, or the nature of the lung space-occupying lesion was to be investigated; (3) the indications for lung resection surgery were clear after clinical judgment; (4) voluntarily completed CPET and signed the informed consent forms before surgery; (5) underwent lung resection for the first time; and (7) complete information.

The exclusion criteria were as follows: (1) individuals with mental disorders and motor disorders; (2) individuals with unstable vital signs before surgery; (3) individuals with various infections, acute metabolic disturbances, or other severe complications before surgery; (4) individuals with a history of cerebral infarction, myocardial infarction, or surgical procedures in the past three months before surgery; (5) individuals diagnosed with malignant tumors other than lung cancer; (6) individuals with absolute contraindications for CPET; and (7) individuals with incomplete information.

The study was approved by the Ethics Committee of the Fourth Hospital of Hebei Medical University (approval number 2025KS101).

### 2.2 Procedures

The personal information and clinical data of the patients according to inclusion criteria were collected. All included patients underwent CPET within one week before surgery. Preoperative transthoracic echocardiography was routinely performed to assess cardiac structure and function. Baseline cardiovascular history, including arrhythmia, coronary heart disease, and deep vein thrombosis, was documented. Patients did not have to discontinue any daily medication before the CPET examination.

CPET was conducted using a MasterScreen-CPX cardiopulmonary exercise assessment system (Jaeger, Germany) in the symptom-limited maximal incremental CPET exercise mode. The following indicators were collected from the CPET examination: reason for termination of exercise; the percentage of maximal workload at termination relative to the predicted value (maximal workload %pred), peak oxygen uptake (peak VO_2_), the percentage of peak oxygen uptake relative to the predicted value (peak VO_2_%pred), the percentage of anaerobic threshold relative to the predicted value (AT %pred), peak heart rate (peak HR), peak metabolic equivalent (peak MET), peak respiratory exchange ratio (peak RER), blood pressure (BP) at rest/peak time, the ventilatory equivalent for carbon dioxide (VE/VCO_2_ slope), and the ventilatory equivalent for carbon dioxide at the anaerobic threshold (VE/VCO_2_@AT).

The indications for immediate termination of exercise were as follows: (1) newly emerging or worsening angina pectoris, or dyspnea; (2) ST segment elevation ≥1.0 mm in leads without diagnostic Q waves (excluding V1 and aVR leads); ST segment depression ≥2.0 mm or downsloping depression during exercise, or significant axis deviation; (3) central nervous system symptoms such as ataxia or dizziness, or signs of impending syncope (presyncope); (4) peripheral hypoperfusion such as cyanosis or pallor; (5) symptoms such as fatigue, shortness of breath, wheezing, leg cramps, or muscle and joint pain that prevent continuation of exercise; (6) a decrease in exercise systolic blood pressure ≥10 mmHg compared to the resting state, or an excessive hypertension response; and (7) severe arrhythmias, including sustained ventricular tachycardia, frequent ventricular premature beats, frequent supraventricular tachycardia, frequent paroxysmal ventricular tachycardia, frequent conduction block, or frequent bradyarrhythmias.

### 2.3 Confirmation of complications

The postoperative complications and follow-up information after discharge of these patients within 30 d postoperatively were collected. The occurrence and prognosis of postoperative cardiopulmonary complications were documented and analyzed, and the correlations between the occurrence of postoperative cardiopulmonary complications and preoperative statistical indicators were investigated.

The cardiorespiratory complications occurring within 30 d postoperatively were recorded. Respiratory complications included: (1) re-intubation or the inability to extubate postoperatively, necessitating transfer to the ICU for mechanical ventilation due to respiratory failure; (2) chest tube duration ≥ 7 d due to prolonged air leak, atelectasis, pleural effusion, etc. (14, 15); (3) pulmonary infection accompanied by changes visible on chest x-ray or chest CT scan; (4) respiratory distress syndrome; and (5) pulmonary embolism. Cardiovascular complications included: (1) myocardial infarction; (2) angina pectoris; (3) heart failure; and (4) arrhythmias requiring treatment. The occurrence of complications was defined as the presence of one or more of the above.

### 2.4 Data analysis

Statistical analysis was conducted using SPSS 27.0 software. Categorical data were expressed as percentages, while quantitative data were expressed as mean ± standard deviation (*x* ± *s*). Chi-square test was employed to compare categorical data. For quantitative data adhering to a normal distribution, independent-sample *t*-test was used for inter-group comparisons. Mann–Whitney *U* test was applied for inter-group comparisons of measurement data that did not follow a normal distribution. The CPET indicators with significant differences were stratified and subjected to logistic regression analysis. Statistical significance was indicated by *P* < 0.05. The sensitivity and specificity of each indicator were also evaluated.

## 3 Results

### 3.1 Clinical data and key CPET indicators

We included a total of 397 patients who met the inclusion criteria and were diagnosed with either lung cancer or pulmonary nodules/pulmonary space-occupying lesions requiring surgical intervention. All patients underwent preoperative CPET. Based on the clinical diagnosis and disease stage, thoracic surgeons performed various types and extents of lung resections: 376 patients underwent video-assisted thoracoscopic lung resection (lung wedge resection/segmentectomy/lobectomy with or without lymph node dissection); 19 patients underwent thoracotomy (e.g., conversion from thoracoscopy to open-chest lung resection and thoracotomy with lung resection and lymph node dissection); and two patients underwent total pneumonectomy combined with lymph node dissection. The 21 patients were excluded due to the small number of patients undergoing thoracotomy or total pneumonectomy. Consequently, 376 patients who underwent video-assisted thoracoscopic lung resection were included in the final statistical analysis. The included cases included 162 males and 214 females with a mean age of 62.5 ± 6.1 years.

The postoperative complications were followed up for 30 d, and 52 patients experienced at least one complication. The incidence of cardiorespiratory complications was 13.8%, with a total of 56 occurrences of seven types of complications. There were no fatalities. The frequency of each type of complication is detailed in Table 1.

**Table 1:** Postoperative complications in patients within 30 d after thoracoscopic lung resection.

Postoperative complications	Patients, *n* (%)
**Prolonged chest tube duration (≥7 d)**	
Atelectasis	7 (1.9)
Pleural effusion	4 (1.1)
Pneumonia	18 (4.8)
Pulmonary embolism	1 (0.3)
Respiratory failure requiring ICU	2 (0.5)
Treated symptomatic cardiac arrhythmia	20 (5.3)
Cardiac ischemia/infarction	4 (1.1)
Cardiac failure	0 (0)
Death	0 (0)

Pneumonia: temperature >38℃, purulent sputum and infiltrate on radiography.

Pulmonary embolism: high probability on ventilation perfusion scan or angiogram.

Comparison between the subgroup without cardiopulmonary complications (NCCP group: 324 cases) and the subgroup with cardiopulmonary complications (CCP group: 52 cases) revealed statistically significant differences in age, extent of lung resection, and lymph node metastasis (*P* < 0.05); no significant differences between the two groups were observed in other general conditions (*P* > 0.05). Regarding the CPET indicators, statistically significant differences were observed in peak HR, peak VO_2_, peak VO_2_%pred, peak MET, and maximal workload %pred between the two groups (*P* < 0.05). In contrast, no statistically significant differences were found in AT %pred, VE/VCO_2_@AT, VE/VCO_2_ slope, and peak RER between the two subgroups (*P* > 0.05). The comparison of general conditions and CPET data between the two subgroups is detailed in Table 2.

**Table 2:** Comparison of general conditions and the core CPET data.

Variables	Total	NCCP	CCP	*P* value
Patients *n* (%)	376 (100)	324 (86.2)	52 (13.8)	
Age (years)	62.5 ± 6.1	61.6 ± 6.0	67.7 ± 4.1	<0.01
Gender				
Male *n* (%)	162 (43.1)	135 (41.7)	27 (51.9)	0.17
Female *n* (%)	214 (56.9)	189 (58.3)	25 (48.1)	
Smoking history				
Yes *n* (%)	105 (27.9)	85 (26.2)	20 (38.5)	0.07
No *n* (%)	271 (72.1)	239 (73.8)	32 (61.5)	
Extent of resection				
WE *n* (%)	150 (39.9)	144 (44.4)	6 (11.5)	<0.01
SE + LE *n* (%)	226 (60.1)	180 (55.6)	46 (88.5)	
Lymph node metastasis				
Yes *n* (%)	25 (6.6)	15 (4.6)	10 (19.2)	<0.01
No *n* (%)	351 (93.4)	309 (95.4)	42 (80.8)	
Histology				
NSCLC *n* (%)	292 (77.7)	253 (78.1)	39 (75.0)	0.71
SCLC *n* (%)	73 (19.4)	61 (18.8)	12 (23.1)	
Benign nodules *n* (%)	11 (2.9)	10 (3.1)	1 (1.9)	
Cardiovascular history				
Arrhythmia *n* (%)	28 (7.4)	21 (6.5)	7 (13.5)	0.08
Coronary heart disease *n* (%)	31 (8.2)	24 (7.4)	7 (13.5)	0.14
Deep vein thrombosis *n* (%)	5 (1.3)	4 (1.2)	1 (1.9)	0.68
LVEF (%)	62.4 ± 6.8	62.7 ± 6.6	60.5 ± 7.8	0.06
CPET indicators				
Peak HR	144.9 ± 18.7	146.8 ± 18.8	133.1 ± 12.7	<0.01
Peak VO_2_ [mL/(min·kg)]	17.9 ± 3.1	18.3 ± 3.1	15.2 ± 1.6	<0.01
Peak VO_2_ (%pred)	77.6 ± 14.0	79.2 ± 14.2	68.2 ± 7.4	<0.01
AT (%pred)	52.8 ± 7.0	54.1 ± 6.3	44.8 ± 5.7	0.64
Peak MET	5.2 ± 0.9	5.3 ± 0.9	4.5 ± 0.6	0.02
VE/VCO_2_@AT	28.2 ± 4.0	27.8 ± 3.9	31.1 ± 3.8	0.94
VE/VCO_2_ slope	28.7 ± 4.0	28.2 ± 3.8	31.9 ± 3.4	0.43
Peak RER	1.3 ± 0.6	1.3 ± 0.7	1.3 ± 0.1	0.59
Maximal workload (%pred)	85.5 ± 18.3	87.1 ± 18.4	75.4 ± 13.8	<0.01

NCCP, no cardiopulmonary complications group; CCP, cardiopulmonary complications group; NSCLC, non-small cell lung cancer; SCLC, small cell lung cancer; WE, lung wedge resection; SE, segmentectomy; LE, lobectomy; LVEF, left ventricular ejection fraction.

### 3.2 CPET indicator thresholds for the prediction of postoperative complications

The indicators exhibiting significant differences between the NCCP and CCP groups following thoracoscopic lung resection were divided into distinct levels. For example, peak VO_2_ was divided into <20 mL/(min·kg), <15 mL/(min·kg); peak VO_2_%pred was divided into <80%, <70%, <60%; peak MET was divided into <6, <5, <4; and maximum workload %pred was divided into <80%, <70%, <60%. The incidence of complications at these indicator levels was then compared between the two subgroups. Chi-square test and logistic regression analysis were performed, and the indicators without statistical significance (*P* > 0.05) were eliminated. The results are shown in Table 3.

**Table 3:** Single-factor logistic regression analysis of postoperative cardiopulmonary complications.

Index	Morbidity	*χ* ^2^	*P* value	*P* value (logistic)
Peak VO_2_ < 20 mL/(min·kg)	51/314	9.3	<0.01	0.015
<15 mL/(min·kg)	25/67	37.7	<0.01	<0.01
Peak VO_2_ (%pred) < 80%	47/213	28.0	<0.01	<0.01
<70%	38/131	38.8	<0.01	<0.01
<60%	8/22	9.9	<0.01	<0.01
Peak MET <6	49/309	6.0	0.014	0.023
<5	40/143	38.7	<0.01	<0.01
<4	11/29	15.3	<0.01	<0.01
Maximal workload (%pred) < 80%	34/154	14.9	<0.01	<0.01
<70%	23/92	12.8	<0.01	<0.01
<60%	7/22	6.3	0.012	0.016

### 3.3 Diagnostic evaluation of CPET indexes

Based on the findings of Section 3.2, the indicator cut-off values resulting in significant differences between the NCCP and CCP groups were analyzed to evaluate the sensitivity, specificity, positive predictive value, and negative predictive value of these parameters at various cut-off levels in predicting postoperative cardiopulmonary complications. As shown in Table 4, the sensitivity and specificity of peak VO_2_%pred <70%, peak MET <5, and maximal workload %pred <80% both exceeded 60%, with the negative predictive values exceeding 90%. The positive predictive values of peak VO_2_ < 15 mL/(min·kg), peak VO_2_%pred <60%, peak MET <4, and maximal workload %pred <60% all exceeded 30%. The diagnostic accuracy and odds ratios (ORs) obtained by categorizing the indicators based on these thresholds are presented in Table 5. The diagnostic accuracy was highest using the following cutoff values of peak VO_2_ [15 mL/(min·kg)], peak VO_2_%pred (60%), peak MET (4), and maximal workload %pred (60%), resulting in ORs of 6.2, 4.0, 4.6, and 3.2, respectively.

**Table 4:** Evaluation of indexes for diagnosis.

Index	Sensitivity (%)	Specificity (%)	Positive predictive value (%)	Negative predictive value (%)
Peak VO_2_ < 20 mL/(min·kg)	98	19	16	98
Peak VO_2_ < 15 mL/(min·kg)	48	87	37	91
Peak VO_2_ (%pred) < 80%	90	49	22	97
Peak VO_2_ (%pred) < 70%	73	71	29	94
Peak VO_2_ (%pred) < 60%	15	95	36	88
Peak MET <6	94	20	16	96
Peak MET <5	77	68	30	95
Peak MET <4	21	94	38	88
Maximal workload (%pred) < 80%	65	63	22	92
Maximal workload (%pred) < 70%	44	79	25	90
Maximal workload (%pred) < 60%	13	95	31	87

**Table 5:** Diagnostic accuracy and the OR values of the main indicators of CPET.

Index	Diagnostic accuracy (%)	OR	95%CI
Peak VO_2_ < 15 mL/(min·kg)	81.6	6.2	3.3–11.7
Peak VO_2_ (%pred) <70%	71.5	6.7	3.5–13.0
Peak VO_2_ (%pred) <60%	84.6	4.0	1.1–6.9
Peak MET <5	69.4	7.2	3.6–14.2
Peak MET <4	84.3	4.6	2.0–10.3
Maximal workload (%pred) <80%	63.3	3.2	1.7–5.9
Maximal workload (%pred) <60%	84.0	3.2	1.2–8.3

OR, odds ratio; CI, confidence interval.

## 4 Discussion

The primary postoperative issues among thoracic surgery patients are cardiorespiratory complications, and the decline in cardiopulmonary function is an important cause of postoperative complications in patients after thoracic surgery. The leading cause of mortality is respiratory failure (16). Cardiorespiratory function mensuration is an essential preoperative examination for thoracic surgery and provides a basis for assessing the risk of anesthesia and postoperative complications.

At rest, the heart and lung functions may have compensatory reserve capacity. The distinction between CPET and traditional heart and lung function tests is that CPET focuses on the interaction between heart and lung functions during exercise. Thus, CPET provides a comprehensive reflection of the body's maximum aerobic metabolic capacity and cardiopulmonary reserve capacity, with a particular emphasis on the simultaneous assessment of heart function and lung function. The oxygen demand of thoracic surgery patients increases by 40%−50% after surgery, requiring higher cardiopulmonary reserves to meet the increase in postoperative oxygen consumption (17–19). CPET monitors various cardiopulmonary variables (e.g., ventilation, metabolic indicators, dynamic blood pressure, heart rate, and electrocardiogram parameters) while stressing the entire cardiopulmonary and oxygen delivery system, thereby evaluating cardiopulmonary function and status simultaneously (20). To some extent, CPET mirrors the stresses placed on the heart and lung functions of thoracic surgery patients, who must cope with the increased metabolic demands induced by surgical trauma. Consequently, CPET provides a comprehensive evaluation of cardiopulmonary reserve capacity and tolerance to pulmonary resection (21). CPET has thus become a recommended method for assessing risks related to anesthesia and surgery and particularly for determining suitability for pulmonary resection and risk of perioperative complications (10, 22).

In this study, the incidence of cardiopulmonary complications after video-assisted thoracoscopic lung resection was 13.8%, lower than that observed in previous studies on traditional thoracotomy and lung resection. This reflects the advantages of thoracoscopic lung resection over traditional methods, including reduced release of inflammatory factors, minimalized functional impact, and shortened postoperative hospital stays. In terms of general patient characteristics, patients in the CCP group were older, had a greater extent of lung resection, and were more likely to have had lymph node dissection compared to patients in the NCCP group, consistent with some previous findings (8, 23, 24). We also found that video-assisted thoracoscopic lung resection posed a relatively low risk, with most complications being mild; however, elderly patients or those undergoing a larger scope of resections were still at high risk for postoperative complications. Our results indicated that several core CPET indicators were significant for predicting the incidence of cardiopulmonary complications following thoracoscopic lung resection (*P* < 0.05): peak HR, peak VO2, peak VO2%pred, MET, and maximal workload %pred. The diagnostic accuracy was highest using the following cut-off values of CPET indicators: peak VO_2_ of 15 mL/(min·kg), peak VO_2_%pred of 60%, peak MET of 4, and maximal workload %pred of 60%.

For pulmonary resection, peak VO_2_ is regarded as the most important indicator for assessing surgical risk. In related studies on pulmonary resection (primarily thoracotomy-based lung resections), patients with peak VO_2_ < 15 mL/(min·kg) were at increased risk of postoperative complications, and peak VO_2_ was the most commonly recommended indicator (20, 22, 25–28). Peak VO_2_%pred is the percentage of a patient's measured peak VO_2_ value relative to the predicted peak VO_2_ value of a healthy individual of the same gender and age. Compared with the matched healthy cohort, a higher peak VO_2_%pred is associated with improved survival and reduced complications (29–32). Our results verify this finding; the risk of postoperative complications was higher for peak VO_2_%pred <60%. However, the threshold of peak VO_2_%pred used to assess the risk of lung resection has not been widely validated, potentially due to the limited generalizability of peak VO_2_%pred values derived from a small matched sample of healthy people (33, 34).

In this study, the peak HR of the CCP subgroup was significantly lower than that of the NCCP subgroup (*P* < 0.05). Heart rate reserve (HRR) serves as an indicator for assessing cardiac function and heart rate adaptability during exercise. However, in this study, we did not compare the HRR inferred from peak HR. The HRR results were biased due to the termination of exercise in some patients during the exercise process due to myocardial ischemia, arrhythmia, and subjective feelings of leg fatigue. Therefore, no statistical analysis of the predictive value of HRR was conducted. Even so, our findings suggest that patients with inadequate cardiac response to increased exercise loads exhibit a higher risk of complications after thoracoscopic lung resection.

We found a correlation between the maximum exercise load and the incidence of postoperative complications, which aligns with previous findings (35). However, no research consensus has been reached on this relationship. Based on our findings, we recommend using peak VO_2_ as the primary CPET indicator complemented with maximal workload %pred and MET for a comprehensive preoperative evaluation of risk factors for cardiopulmonary complications. Patients identified as high-risk [e.g., peak VO_2_ < 15 mL/(min·kg) or peak VO_2_%pred <60%] may benefit from enhanced postoperative monitoring in intensive care units, routine assessment of cardiac biomarkers such as troponin and pro-BNP, and careful reconsideration of surgical indications.

Although lower AT is associated with higher morbidity and mortality in abdominal, vascular, transplant and bariatric surgeries (36), we did not find a similar correlation for pulmonary resection. In our study, VE/VCO_2_@AT and VE/VCO_2_ slope were higher in the postoperative CCP subgroup than in the NCCP subgroup, although the differences were not statistically significant (*P* > 0.05). Recent studies have suggested that VE/VCO_2_ slope can predict postoperative cardiopulmonary complications and mortality (37) and can be regarded as an independent predictor of postoperative mortality (38–40). Our study failed to confirm this relationship point due to several limitations: the relatively good general condition of the enrolled population, the relatively small scope of surgical resection, and the low mortality rate (no deaths occurred within 30 d in our enrolled population). Further validation should be conducted in a broader population with an extended follow-up period.

The spirometry and diffusion capacity of the lung for carbon monoxide are almost universally used as important indicators by thoracic surgeons. Patients whose test results fall below the threshold values are further assessed by low-tech exercise tests (41); however, CPET is still considered as the gold standard for evaluating cardiopulmonary metabolic function and predicting the risks associated with pulmonary resection (11–13, 42–44). However, Clark et al. found that few thoracic surgeons strictly complied with American College of Chest Physicians guidelines (45).

Additionally, it is worth noting that arrhythmia and pulmonary infection were the most common postoperative complications in our study. Thus, even though more serious cardiopulmonary complications attract the most attention, the potential risks of arrhythmia and pulmonary infection cannot be ignored, especially for high-risk patients. In these cases, it is important to prepare a response plan for cardiopulmonary complications in advance.

### 4.1 Limitations of the study

Early screening for lung cancer enables early lesion detection, thereby reducing the scope of surgical resection and allowing the application of thoracoscopic surgical approaches. Thus, early screening significantly reduces the occurrence of postoperative complications and allows some elderly lung cancer patients who cannot tolerate thoracotomy to receive radical treatment. Due to the limited number of cases with large resection scope and thoracotomy, these patients were excluded from the study, leading to a certain bias. In addition, the overall condition and cardiopulmonary function of the enrolled patients in this study were generally good. The conditions of some patients were classified as contraindications because of old age, poor general condition, and poor cardiac and/or pulmonary function with obvious clinical symptoms. Others declined testing because they were unable to cooperate. As a result, few patients with poor CPET indicators were included. Intraoperative variables such as operative duration and intraoperative complications were not included in the analysis. These factors may independently contribute to postoperative complications and should be considered in future studies. Future research is needed that includes more patients with large lung resection scopes and poor cardiopulmonary function, providing a more comprehensive reference for the prediction of complications after lung resection.

## 5 Conclusion

The results of this study suggest that certain CPET-based preoperative indicators can predict the risk of postoperative complications among patients with generally good cardiopulmonary function and relatively mild postoperative complications. Patients with peak VO_2_ < 15 mL/(min·kg) or peak VO_2_%pred <60% during preoperative CPET examination were found to be at increased risk of postoperative complications. These indicators can be used for the preoperative stratification of patients scheduled for video-assisted thoracoscopic lung resection. Combining these indicators with maximal workload %pred and MET during pre-surgical assessments can help thoracic surgeons formulate preventive measures or prepare for potential postoperative complications.

## Data Availability

The raw data supporting the conclusions of this article will be made available by the authors, without undue reservation.
